# Genes responsive to both oxidant stress and loss of estrogen receptor function identify a poor prognosis group of estrogen receptor positive primary breast cancers

**DOI:** 10.1186/bcr2120

**Published:** 2008-07-17

**Authors:** Christina Yau, Christopher C Benz

**Affiliations:** 1 Buck Institute for Age Research, Redwood Boulevard, Novato, California 94945, USA

## Abstract

**Introduction:**

Oxidative stress can modify estrogen receptor (ER) structure and function, including induction of progesterone receptor (PR), altering the biology and clinical behavior of endocrine responsive (ER-positive) breast cancer.

**Methods:**

To investigate the impact of oxidative stress on estrogen/ER-regulated gene expression, RNA was extracted from ER-positive/PR-positive MCF7 breast cancer cells after 72 hours of estrogen deprivation, small-interfering RNA knockdown of ER-α, short-term (8 hours) exposure to various oxidant stresses (diamide, hydrogen peroxide, and menadione), or simultaneous ER-α knockdown and oxidant stress. RNA samples were analyzed by high-throughput expression microarray (Affymetrix), and significance analysis of microarrays was used to define gene signatures responsive to estrogen/ER regulation and oxidative stress. To explore the association of these signatures with breast cancer biology, microarray data were analyzed from 394 ER-positive primary human breast cancers pooled from three independent studies. In particular, an oxidant-sensitive estrogen/ER-responsive gene signature (Ox-E/ER) was correlated with breast cancer clinical parameters and disease-specific patient survival (DSS).

**Results:**

From 891 estrogen/ER-regulated probes, a core set of 75 probes (62 unique genes) responsive to all three oxidants were selected (Ox-E/ER signature). Ingenuity pathway analysis of this signature highlighted networks involved in development, cancer, and cell motility, with intersecting nodes at growth factors (platelet-derived growth factor-BB, transforming growth factor-β), a proinflammatory cytokine (tumor necrosis factor), and matrix metalloproteinase-2. Evaluation of the 394 ER-positive primary breast cancers demonstrated that Ox-E/ER index values correlated negatively with PR mRNA levels (*r*_p _= -0.2; *P *= 0.00011) and positively with tumor grade (*r*_p _= 0.2; *P *= 9.741 × e^-5^), and were significantly higher in ER-positive/PR-negative versus ER-positive/PR-positive breast cancers (*t*-test, *P *= 0.0008). Regardless of PR status, the Ox-E/ER index associated with reduced DSS (*n* = 201; univariate Cox, *P *= 0.078) and, using the optimized cut-point, separated ER-positive cases into two significantly different DSS groups (log rank, *P *= 0.0009).

**Conclusion:**

An oxidant-sensitive subset of estrogen/ER-responsive breast cancer genes linked to cell growth and invasion pathways was identified and associated with loss of PR and earlier disease-specific mortality, suggesting that oxidative stress contributes to the development of an aggressive subset of primary ER-positive breast cancers.

## Introduction

Estrogen receptor (ER; α isoform) is a redox-sensitive transcription factor, and breast cancer co-expression of progesterone receptor (PR) has long been clinically used to signify a functioning ER response pathway [1] and identify breast cancers that are most likely to respond to ER-targeted endocrine therapy [2-4]. Endocrine therapy, in turn, can alter tumor expression of ER and PR [5,6]; in particular, upon acquiring resistance to an endocrine agent such as tamoxifen, metastatic breast cancers usually retain ER expression [5] but frequently exhibit loss of PR expression [6]. At diagnosis, ER-positive/PR-negative breast cancers appear to be less responsive to endocrine therapy and associated with earlier metastatic relapse than ER-positive/PR-positive cases [2]. Despite the clinical biomarker utility of PR in conjunction with ER, factors that determine the altered biology and more aggressive clinical nature of ER-positive/PR-negative breast cancers remain unclear, with aging [7], activated growth factor receptor signaling [8-11], and oxidative stress [7] all implicated in the loss of PR expression.

As a putative etiologic factor for both aging and age-related diseases, oxidative stress is an attractive mechanistic hypothesis for the biological heterogeneity of ER-positive breast cancers, including PR status. Reactive oxygen species (ROS) are critical mediators of growth factor receptor signaling [12] and estrogen-inducible cell proliferation [13,14]. Not only has the carcinogenic potential of estrogen exposure been attributed to its oxidation chemistry [15,16], but oxidative stress pathways activated during cell immortalization and transformation have been correlated with breast cancer clinical prognosis [17].

Two major cellular consequences of excess oxidant exposure can specifically influence ER pathways and the endocrine responsiveness of ER-positive breast cancer. The first of these is that oxidative stress can reversibly or irreversibly directly alter protein structure. Among intracellular proteins most sensitive to oxidant-induced structural and functional damage are redox-sensitive zinc finger transcription factors such as ER [18] and Sp1 [19], whose zinc finger cysteine residues are readily oxidized, eliminating their DNA-binding activity. In ER-positive breast cancers, loss of Sp1 DNA binding activity has been correlated with aging in association with increased tumor content of the oxidative stress marker P-Erk5 [7]. Although not necessarily associated with aging, loss of ER DNA-binding activity has been found in up to one-third of all ER-positive breast cancers and correlated with loss of PR expression [20]. Because both ER-α and Sp1 DNA-binding and transactivating functions are needed for optimal estrogenic stimulation of genes such as PR and Bcl2, ER-positive breast cancers subjected to sufficient oxidative stress would be expected to exhibit suppressed expression of these estrogen-inducible genes.

A second major consequence of oxidative stress is its association with kinase-dependent signaling pathways. Aside from mediating growth factor receptor signaling, ROS directly inhibit protein tyrosine phosphatases and enhance Src, c-Jun amino-terminal kinase (JAK), Ras, protein kinase C (PKC), and mitogen-activated protein kinase (MAPK) signal transduction [21]. These activated kinase pathways also modulate ER signaling [22] and have been implicated in endocrine resistance [23-26]. In experimental models, excess MAPK signaling has been shown to repress ER expression and impair estrogen-inducible gene transcription [27]; and expression microarray analyses have shown that activation of MAPK signaling in cultured ER-positive breast cancer cells induces a profile of gene expression similar to that of ER-negative breast cancer [28]. Thus, ROS-activated kinases represent another means by which ER function can be compromised after oxidant stress and potentially associated with the clinical phenotype of ER-positive/PR-negative breast cancer.

Although gene expression profiles signifying estrogen responsiveness [29] and oxidative stress [30] have been independently identified from studies of ER-positive breast cancer cell line models, altered ER-regulated gene expression within the context of oxidative stress has not yet been addressed. Likewise, models simulating loss of estrogen-induced gene expression, as expected with aging and menopause, in contrast to estrogen-stimulated gene expression, have not been experimentally explored. In the present study, both estrogen deprivation and small-interfering RNA (siRNA) knockdown of ER-α are utilized to identify a complete set of estrogen/ER-regulated genes from which a subset responsive to all three forms of oxidant stress (diamide, hydrogen peroxide, and menadione) are derived as an oxidant-sensitive estrogen/ER gene signature (Ox-E/ER). Also, oxidant-sensitive genes experimentally determined following ER knockdown are analyzed in relation to estrogen/ER-regulated genes to derive an alternative Ox-E/ER signature (Ox'-E/ER). The relationships of these indicators of oxidant exposure, in particular the Ox-E/ER gene signature, to breast cancer biology and clinical outcome are further explored using microarray data from 394 ER-positive primary human breast cancers pooled from three independently published studies.

## Materials and methods

### Cells, culture treatments, and RNA extraction

ER-positive/PR-positive MCF7 human breast cancer cells were obtained from the American Type Culture Collection (Rockville, MD, USA). Cells were routinely maintained at 37°C and 5% carbon dioxide in Dulbecco's modified Eagle's medium (DMEM) supplemented with 10% fetal bovine serum, 1% penicillin-streptomycin, and 10 ng/ml insulin. Media and supplements were purchased from Mediatech, Inc. (Herndon, MA, USA). For ER-α knockdown, MCF7 cells were transfected with 100 nmol/l anti-ER-α siRNA (Dharmacon, IL, USA) using Lipofectamine-2000 (Invitrogen, CA, USA), in accordance with the manufacturer's protocol, and incubated for 72 hours. For estrogen deprivation, cells were plated in DMEM and allowed to attach overnight. The media were then changed to DMEM without phenol red; supplemented with 10% charcoal-stripped fetal bovine serum, 1% penicillin-streptomycin, and 10 ng/ml insulin; and cultures were incubated for an additional 72 hours. For oxidant treatments, MCF7 cells were treated with 275 nmol/l diamide or 0.5 mmol/l hydrogen peroxide (H_2_O_2_) or 10 μmol/l menadione for 8 hours. These oxidant doses and exposure times were chosen for their ability to significantly impair ER transcriptional activation without significantly impairing total ER content and cell viability (Additional file 1). Diamide and menadione were purchased from Sigma (Louisville, MO, USA) and H_2_O_2 _was obtained from VWR International (West Chester, PA, USA). For simultaneous ER-α knockdown and oxidant stress, MCF7 cells were transfected with 100 nmol/l anti-ER-α siRNA (Dharmacon, IL, USA) using Lipofectamine-2000 (Invitrogen, CA, USA), in accordance with the manufacturer's protocol, and incubated for 64 hours before oxidant treatment (275 nmol/l diamide or 0.5 mmol/l H_2_O_2 _or 10 μmol/l menadione) for 8 hours. All treatments were performed in duplicate. Total cell RNA was extracted using RNeasy Mini Kit in accordance with the manufacturer's instruction (Qiagen, CA, USA). RNA was diluted to 0.4 μg/μl, and RNA integrity was assayed using a 2100 Bioanalyzer (Agilent, CA, USA).

### Microarray analysis and gene signature derivations

Total RNA (4 μg per MCF7 sample) was labeled and analyzed using Affymetrix, Inc. (Santa Clara, CA, USA) HT-HG_U133A Early Access Arrays containing 22.9 K probes representing about 13 K unique Unigenes. Analyses were performed by standard Affymetrix procedures within the Lawrence Berkeley National Laboratory and Life Science Divison's Molecular Profiling Laboratory. Probe set measurements were generated from quantified Affymetrix image (.CEL) files using the RMA algorithm in Bioconductor R [31]. MCF7 microarray data were deposited into the public Gene Expression Omnibus database (GSE10061). Significance analysis of microarrays software [32] was used to determine significant changes in gene expression between treated and corresponding reference samples (control MCF7 for the single agent treatments, and ER-α knockdown MCF7 for the combined knockdown and oxidant stress treatment conditions). Venn diagrams were constructed in Bioconductor R, and the overlapping probes between treatment conditions were used to define the following gene signatures: estrogen/ER genes (overlapping probes from estrogen deprivation and ER-α siRNA treatment), Ox genes (probes commonly affected by all three oxidant stresses in control MCF7), Ox' genes (probes commonly affected by all three oxidant stresses after ER-α knockdown in MCF7), Ox-E/ER genes (estrogen/ER genes responsive to all three oxidant stresses in control MCF7), and Ox'-E/ER genes (estrogen/ER genes responsive to all three oxidant stresses following ER-α knockdown).

### Gene signatures and clinical parameters for 394 estrogen receptor positive primary breast tumors

Our own tumor dataset consisted of 102 early-stage age-stratified ER-positive primary breast cancer samples; these samples were obtained following multi-institutional review board approval and all information pertaining to these samples, their RNA processing, and microarray analysis has previously been reported [33], with primary data deposited into the Gene Expression Omnibus database (GSE7378 and GSE8193). Two other publicly available datasets were also used in this analysis: 214 ER-positive cases were selected from the study conducted by Miller and coworkers [34], and 79 ER-positive cases were selected from the study conducted by Hess and colleagues [35], resulting in a pooled set of 394 ER-positive primary breast tumors. Although treatment data were not available for all samples, more than 50% of these 394 cases received adjuvant systemic therapy. Because all three microarray studies used similar Affymetrix U133A platforms, all probe set measurements were generated using the RMA algorithm in Bioconductor R and related by Affymetrix probe identifiers. Individual datasets were mean-centered, and in order to remove possible systematic biases based on data source, distance-weighted discrimination [36] (DWD) was applied. ERBB2 status was reported for 181 cases in the pooled 394 ER-positive breast cancer dataset. An expression array cut-off value for ERBB2 probe 216836_s_at, previously shown to accurately reflect clinical ERBB2 status [37], was determined by minimizing misclassification error among the 181 samples with known ERBB2 status; and the remaining 213 cases were assigned ERBB2 status based on the expression array cut-off value.

Gene set enrichment analysis (GSEA) was performed using GSEA software version 2.1 [38] to assess all gene signatures with respect to clinical parameters. Ranking of genes with respect to categorical tumor phenotypes such as PR status, expression array determined ERBB2 status, nodal status, and grade was based on the signal-to-noise metric; and Pearson correlation was used to rank-order genes with respect to age at diagnosis. For genes with multiple probes, the median value from all expressed probes was used. Each gene signature enrichment score was derived as a function of the likelihood of that signature set being among the most highly ranked genes for each tumor phenotype, and enrichment score significance was estimated using 1,000 random permutations of the sample labels in the tumor dataset. For comparison, enrichment of four other reported gene signatures was also assessed; these included MAPK [28], proliferation [39], luminal [40], and sustained estrogen-induced [29] gene signatures, as listed in Additional file 2. To explore the relationship between oxidative stress and proliferation, hierarchical clustering of the combined dataset was performed based on expression of the proliferation signature [39] using Cluster software [41] and visualized with Java TreeView [42]. The dataset was dichotomized into tumors with higher proliferation signature (H) and tumors with lower proliferation signature (L). Numeric indices were calculated for the Ox, Ox', Ox-E/ER and Ox'-E/ER signatures as follows:

$$\text{Index}=\sum_{\text{j}\in \text{U}}{\text{x}}_{\text{j}}/\text{n}-\sum_{\text{j}\in \text{D}}{\text{x}}_{\text{j}}/\text{m}$$

Where x is the DWD-transformed expression measure, j is a gene indicator, ∈ signifies 'contained in', and U and D are the set of probes upregulated and downregulated by oxidative stress, respectively. A two-sample *t*-test with unequal variance was used to compare index values for each signature with respect to tumor proliferation status (H, L).

### Ox-E/ER gene signature functional analysis and prognostic validation

Ingenuity pathway analysis software (Ingenuity Systems Inc., Mountain View, CA, USA) was used to identify top biological networks represented by the Ox-E/ER gene signature. A two-sample *t*-test with unequal variance was used to compare Ox-E/ER index values with respect to PR status, array determined ERBB2 status, grade, and nodal status. Pearson correlations were determined between the index and PR mRNA expression level as well as each clinical parameter (age, array determined ERBB2 status, grade and nodal status). The pooled tumor sample set contained 201 cases with disease-specific survival (DSS) information (>33% adjuvant treated), and 263 cases with relapse-free survival (RFS) information (>46% adjuvant treated); and the prognostic value of the gene signature index was evaluated by univariate Cox analysis. Univariate Cox analyses based on patient age, tumor PR status, PR mRNA level, nodal status, and grade were also performed. Multivariate Cox analysis was used to determine which of these variables demonstrated independent prognostic significance. For the 201 cases with DSS data, an optimized cut-point for the Ox-E/ER gene signature index was determined using a modified log-rank statistic [43]. Kaplan-Meier analyses were performed on this dataset dichotomized by the optimized index cut-point and compared with the same data dichotomized by PR status.

## Results

### Defining gene signatures responsive to estrogen/estrogen receptor modulation and oxidative stress

**Figure 1:**
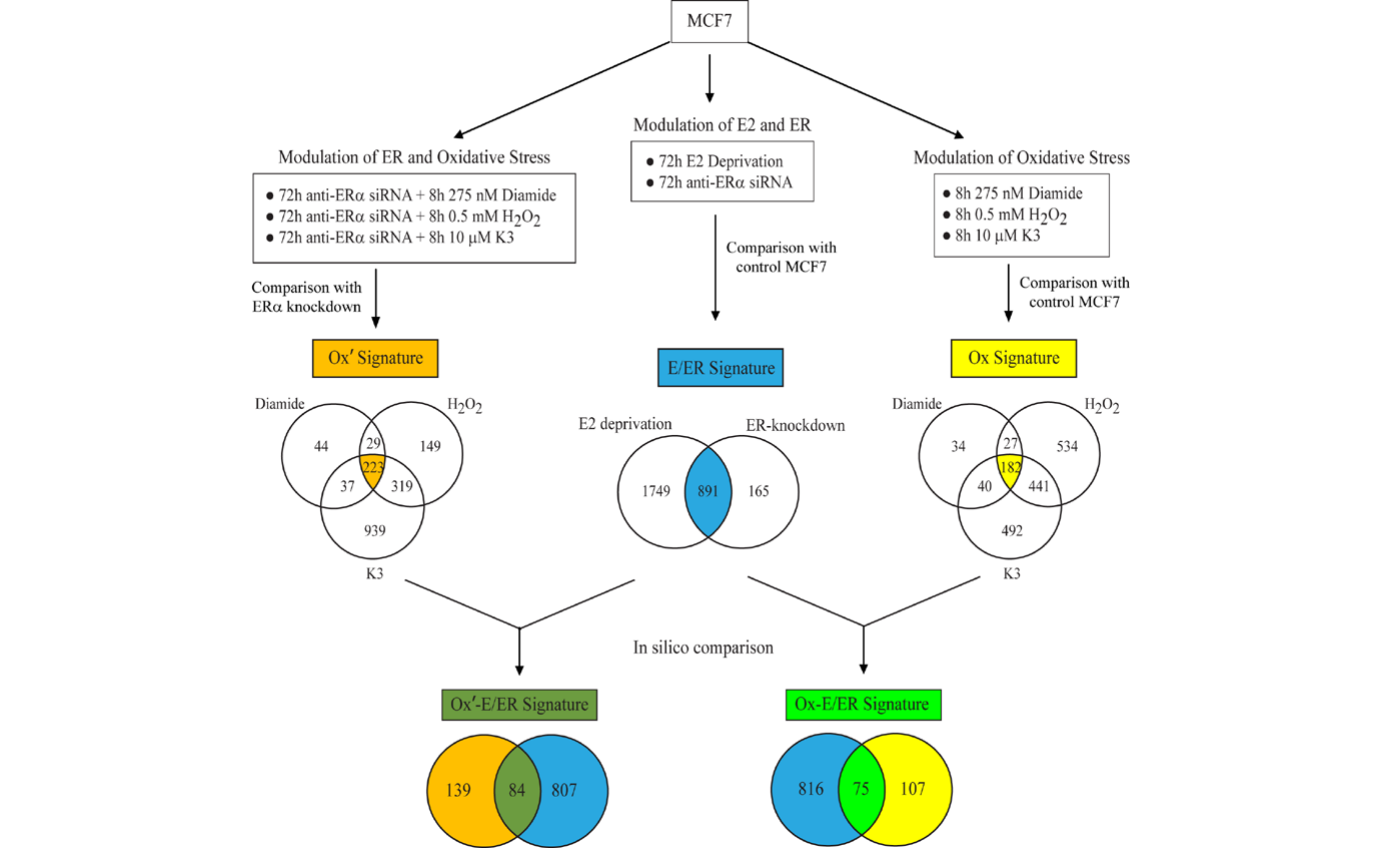
Experimental conditions and derivation of gene signatures based on MCF7 treatments and microarray analyses. Depicted by the left set of conditions, the Ox' gene signature (color-coded in gold) was derived as the overlapping probe set after significance analysis of microarrays (SAM) comparisons of expression microarrays results from MCF7 treated with both estrogen receptor (ER)-α small-interfering RNA (siRNA) and oxidants, relative to MCF7 treated only with ER-α siRNA. Depicted by the middle set of conditions, the estrogen (E)/ER gene signature (color-coded in blue) was derived as the overlapping set of differentially expressed MCF7 probes following estradiol (E_2_) deprivation and ER-α knockdown. Depicted by the right set of conditions, the Ox signature (color-coded yellow) was derived as the overlapping probe set after SAM comparisons of microarray results from oxidant stressed MCF7 relative to control MCF7. The overlap between the Ox' and E/ER signatures defines the Ox'-E/ER signature (color-coded dark green); and the overlap between the Ox and E/ER signatures defines the Ox-E/ER gene signature (color-coded light green).

As shown in Figure 1, differential expression analysis identified 2,640 probes as estrogen responsive, based on 72 hours of MCF7 E deprivation (1.5× fold change, false discovery rate q = 0.01); similarly, 1,056 differentially expressed probes were considered to be ER regulated, based on 72 hours of knockdown of ER-α in MCF7 by siRNA. Altogether, an overlapping set of 891 probes was identified as estrogen/ER responsive and evaluated further with respect to their oxidant sensitivity (Figure 1).

Relative to a recent microarray study of estrogen-treated MCF7 [29], only 31% of these 891 probes presently identified as responsive to either estrogen deprivation or ER-α knockdown are also stimulated by estrogen treatment of MCF7 cells. With regard to MCF7 oxidant sensitivity, Figure 1 shows that 283 probes were identified as differentially expressed after 8 hours of diamide treatment, whereas 1,184 and 1,155 probes exhibited significant changes in expression after 8 hours of treatment with H_2_O_2 _or menadione, respectively. Altogether, 1,750 probes exhibited some oxidant sensitivity, and 182 probes commonly affected by all three oxidants defined the Ox gene signature (Figure 1). Of these, a set of 75 probes (62 unique genes) commonly affected by all oxidants and representing only 8% of all E/ER responsive probes defined the Ox-E/ER gene signature (Figure 1). Within this Ox-E/ER gene signature is the well characterized estrogen responsive gene Bcl2. PR and GREB1, two genes commonly associated with estrogen responsiveness in breast cancer, are not included in the signature because their expression levels were significantly reduced by H_2_O_2 _and menadione treatments but not significantly reduced by the thiol-specific oxidant diamide. Following ER-α knockdown in MCF7, expression of 333 probes was subsequently altered by 8 hours of diamide, whereas levels of 720 and 1,518 probes were subsequently altered by H_2_O_2 _or menadione treatments, respectively (Figure 1). Among the total 1,740 oxidant affected probes in MCF7 with downregulated ER-α, 223 were commonly affected and defined the Ox' gene signature (Figure 1). Among this Ox' gene signature are 84 estrogen/ER-responsive probes, which defined the Ox'-E/ER gene signature (Figure 1). There is a 33-probe overlap (25 unique genes) between the Ox-E/ER and Ox'-E/ER gene signatures. Genes responsive to each of the eight different treatment conditions schematically illustrated in Figure 1 are fully listed in Additional file 3.

### Gene signature enrichment and estrogen receptor-positive breast cancer clinical features

The pooled clinical dataset of 394 ER-positive primary breast cancers were subjected to GSEA to identify clinical parameters (patient age at diagnosis, tumor grade, PR, ERBB2, and nodal status) potentially associated with the five derived gene signatures, as well as previously reported gene signatures representing sustained estrogen induction [29], luminal subtype [40], MAPK activation [28], and tumor proliferation [39]. The results of these analyses are summarized in Tables 1 and 2, and include values for the normalized enrichment score, nominal and adjusted (family-wise error rate [FWER]) *P *values.

**Table 1:** Gene set enrichment analysis of 394 ER-positive breast cancers: part 1

		Comparison								
		
		PR^- ^over PR^+^			ERBB2^- ^over ERBB2^+^			Age		
		
	Gene set	NES	Nominal *P*	FWER *P*	NES	Nominal *P*	FWER *P*	NES	Nominal *P*	FWER *P*
Modulation of E_2 _and ER	Estrogen/ER induced	-1.03	0.42	0.91	1.06	0.36	0.87	**-1.60**	**0.02**	**0.18**
	Estrogen/ER repressed	-1.14	0.27	0.81	-0.99	0.46	0.90	**1.67**	**0.01**	**0.11**
Modulation of oxidative stress	Ox induced	1.15	0.28	0.78	**-1.61**	**0.04**	**0.19**	-1.45	0.07	0.36
	Ox repressed	**-1.53**	**0.02**	**0.24**	1.19	0.20	0.75	1.18	0.21	0.74
	Ox-E/ER Ox-induced	0.90	0.54	0.95	-1.43	0.11	0.40	-1.45	0.09	0.37
	Ox-E/ER Ox repressed	**-1.75**	**0.00**	**0.07**	1.36	0.11	0.53	-1.13	0.29	0.81
Modulation of ER and oxidative stress	Ox' induced	1.17	0.24	0.75	**-1.66**	**0.02**	**0.13**	-1.21	0.19	0.71
	Ox' repressed	**-1.54**	**0.04**	**0.23**	1.21	0.21	0.73	**1.71**	**0.01**	**0.09**
	Ox'-E/ER Ox induced	-1.30	0.15	0.61	-1.04	0.40	0.86	-1.19	0.24	0.75
	Ox'-E/ER Ox repressed	-1.39	0.11	0.44	0.94	0.54	0.93	1.49	0.07	0.32
Literature^a^	MAPK induced	1.16	0.28	0.77	-0.98	0.48	0.91	-0.74	0.78	0.98
	MAPK repressed	-1.17	0.25	0.77	1.09	0.33	0.83	0.99	0.46	0.92
	Luminal markers	**-1.69**	**0.03**	**0.10**	**1.68**	**0.02**	**0.11**	**1.85**	**0.00**	**0.01**
	Sustained estrogen-induced	1.00	0.45	0.90	-1.26	0.18	0.64	-1.07	0.36	0.85
	Proliferation markers	**1.92**	**0.00**	**0.01**	**-1.95**	**0.00**	**0.01**	**-1.70**	**0.04**	**0.09**

Gene sets showing nominal enrichment (*P *< 0.05) are denoted in bold. ^a^Mitogen-activated protein kinase (MAPK) [42], luminal [54], sustained estrogen-induced [43], and proliferation signatures [53] as shown in Additional file 2. E_2_, estradiol; ER, estrogen receptor; FWER, family-wise error rate; NES, normalized enrichment score; Ox-E/ER, oxidant-sensitive estrogen/ER gene signature; PR, progesterone receptor.

**Table 2:** Gene set enrichment analysis of 394 ER-positive breast cancers: part 2

		Comparison											
		
		LN^- ^over LN^+^			Grade 3 over grade 1			Grade 3 over grade 2			Grade 2 over grade 1		
		
	Gene set	NES	Nominal *P*	FWER *P*	NES	Nominal *P*	FWER *P*	NES	Nominal *P*	FWER *P*	NES	Nominal *P*	FWER *P*
Modulation of E_2 _and ER	Estrogen/ER induced	-1.38	0.07	0.47	1.40	0.07	0.45	1.14	0.27	0.78	1.27	0.15	0.63
	Estrogen/ER repressed	1.35	0.11	0.52	**-1.51**	**0.05**	**0.28**	-1.27	0.16	0.62	-1.21	0.18	0.69
Modulation of oxidative stress	Ox induced	1.14	0.30	0.79	1.44	0.08	0.38	1.55	0.06	0.23	0.78	0.75	0.98
	Ox repressed	1.03	0.42	0.87	-1.32	0.11	0.57	**-1.51**	**0.03**	**0.29**	-0.88	0.65	0.95
	Ox-E/ER Ox induced	1.10	0.36	0.82	1.11	0.34	0.80	1.11	0.35	0.82	-0.98	0.46	0.90
	Ox-E/ER Ox repressed	1.24	0.20	0.69	-1.00	0.44	0.90	-1.34	0.12	0.54	-0.42	1.00	1.00
Modulation of ER and oxidative stress	Ox' induced	1.00	0.44	0.89	**1.50**	**0.05**	**0.30**	1.53	0.06	0.25	0.82	0.70	0.97
	Ox' repressed	0.99	0.47	0.90	**-1.74**	**0.01**	**0.06**	**-1.70**	**0.01**	**0.09**	-1.19	0.21	0.73
	Ox'-E/ER Ox induced	1.03	0.42	0.87	0.91	0.52	0.93	0.75	0.77	0.98	-1.43	0.12	0.40
	Ox'-E/ER Ox repressed	1.24	0.20	0.68	**-1.65**	**0.02**	**0.12**	-1.35	0.13	0.53	-1.48	0.06	0.32
Literature^a^	MAPK induced	0.99	0.45	0.90	0.81	0.70	0.98	0.95	0.52	0.92	-0.55	0.96	1.00
	MAPK repressed	-1.09	0.31	0.81	1.06	0.38	0.84	-1.02	0.44	0.88	1.17	0.27	0.75
	Luminal markers	1.19	0.32	0.73	**-1.71**	**0.02**	**0.08**	**-1.77**	**0.01**	**0.05**	-0.81	0.65	0.97
	Sustained estrogen-induced	-1.20	0.23	0.69	**1.79**	**0.00**	**0.03**	1.33	0.11	0.54	**1.63**	**0.02**	**0.14**
	Proliferation markers	**-1.76**	**0.02**	**0.06**	**2.02**	**0.00**	**0.00**	**1.97**	**0.00**	**0.00**	**1.90**	**0.00**	**0.01**

Gene sets showing nominal enrichment (p < 0.05) are denoted in bold.

Gene sets showing nominal enrichment (*P *< 0.05) are denoted in bold. ^a^Mitogen-activated protein kinase (MAPK) [42], luminal [54], sustained estrogen-induced [43], and proliferation signatures [53] as shown in Additional file 2. E_2_, estradiol; ER, estrogen receptor; FWER, family-wise error rate; LN, lymph node; NES, normalized enrichment score; Ox-E/ER, oxidant-sensitive estrogen/ER gene signature; PR, progesterone receptor.

The luminal signature, as well as the Ox, Ox', and Ox-E/ER genes repressed by oxidant stress, showed nominal enrichment in ER-positive/PR-positive tumors (or suppression in ER-positive/PR-negative tumors), whereas the proliferation signature showed nominal enrichment in ER-positive/PR-negative tumors (*P *< 0.05). However, after adjustment for multiple gene set testing, only enrichment of the proliferation signature in ER-positive/PR-negative primary breast cancers retained significance (FWER, *P *= 0.01). A strong trend was observed for enrichment of oxidant-repressed Ox-E/ER genes (such as Bcl2) in ER-positive/PR-positive tumors, reflecting their suppression in ER-positive/PR-negative tumors (FWER, *P *= 0.07). Also, the proliferation signature, along with the Ox and Ox' genes induced by oxidative stress, exhibited nominal enrichment in ERBB2 over-expressing tumors, whereas the luminal signature was nominally enriched in ERBB2-negative tumors (*P *< 0.05). However, after multiple gene set testing, only enrichment of the proliferation signature retained significance in the ERBB2 over-expressing tumors (FWER, *P *= 0.01).

With regard to breast cancer age at diagnosis, GSEA indicated that estrogen/ER-repressed genes were positively correlated whereas estrogen/ER-stimulated genes were negatively correlated with older age at diagnosis (nominal *P *< 0.05), a finding consistent with the previously reported loss of estrogen-induced gene expression in ER-positive breast cancers arising later in life [29]. Also consistent with previous age-linked breast cancer features [33] was the observed negative correlation between the proliferation signature and increasing age at diagnosis (Table 1) and the enriched expression of luminal signature in ER-positive breast cancers arising later in life (nominal *P *< 0.05). The repressed Ox' genes (suggesting decreased oxidative stress) also appeared to be enriched among tumors arising later in life (nominal *P *< 0.05); and consistent with this observation was the trend for Ox and Ox-E/ER genes induced by oxidative stress to exhibit lower expression with increasing age at diagnosis (nominal *P *= 0.07 and 0.09, respectively). After correcting for multiple gene-set testing, however, only the association between the luminal signature and older age at diagnosis retained significance (FWER, *P *= 0.01).

Among the 15 gene sets analyzed in Table 2, only the proliferation signature showed significant enrichment with respect to regional lymph node involvement, with node-positive tumors showing significantly higher expression of the proliferation markers (nominal *P *= 0.008; FWER, *P *= 0.02). There was also significant enrichment of the proliferation signature with ER-positive tumors of increasing grade in all pair-wise comparisons. In contrast, luminal markers were nominally enriched in grade 1 and 2 tumors over grade 3 tumors, whereas the previously reported estrogen-induced gene signature was enriched in grade 2 and 3 tumors over their grade 1 counterparts. There was also increased enrichment of Ox and Ox' repressed gene expression in grade 2 over grade 3 tumors (nominal *P *= 0.03 and 0.01, respectively), and Ox' repressed gene expression was nominally higher in grade 1 over grade 3 tumors, with reciprocal enrichment of Ox' induced genes in grade 3 over grade 1 tumors. No significant associations with respect to tumor grade were observed in other pair-wise comparisons; in particular, the Ox-E/ER signature did not show any significant grade-associated enrichment.

### Oxidative stress associations with tumor proliferation

**Figure 2:**
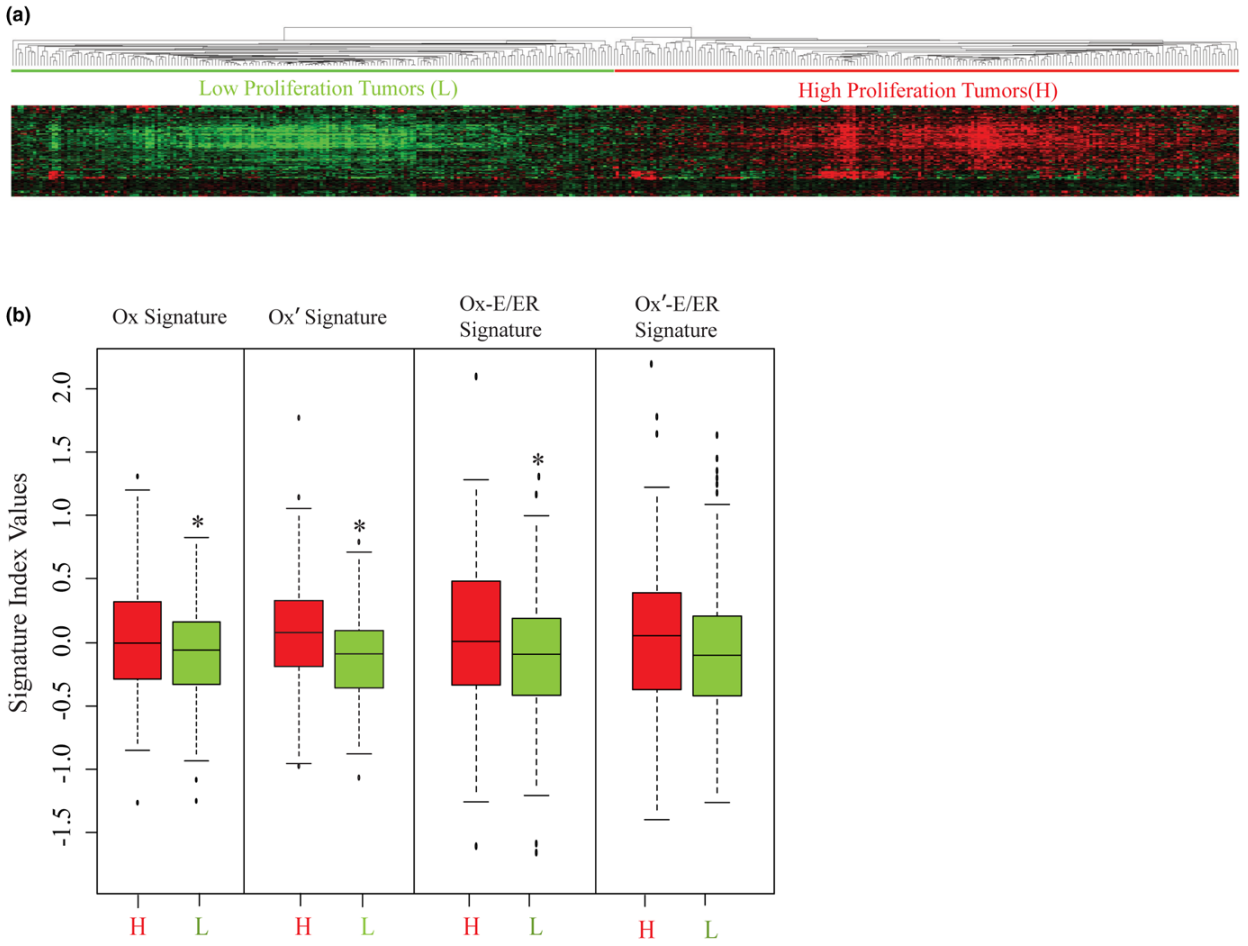
Relationship between oxidative stress and ER-positive tumor proliferation. **(a) **Unsupervised clustering of 394 estrogen receptor (ER)-positive breast cancers based only on expression of the 71-gene proliferation signature, revealing two major clusters of tumors showing high (H) and low (L) expression of the proliferation genes. **(b) **Box plots showing the expression indices determined for each of the Ox, Ox', Ox-E/ER, and Ox'-E/ER signatures in tumors identified from (panel a) as having either high (H) or low (L) expression of the proliferation genes. *Statistically significant difference in mean expression index (*t*-test *P *< 0.05). Ox-E/ER, oxidant-sensitive estrogen/ER gene signature.

Indices determined from each of the Ox, Ox', Ox-E/ER, and Ox'-E/ER signatures were correlated with ER-positive tumor proliferation status. As shown in Figure 2a, when proliferation markers [39] were used to perform unsupervised hierarchical clustering of the 394 pooled ER-positive breast cancer cases, two comparably sized subsets were identified (H, L). The subset with higher (H) expression of proliferation genes was associated with significantly higher Ox, Ox', and Ox-E/ER indices (*t*-test, *P *< 0.05) relative to the subset with lower (L) expression of proliferation genes (Figure 2b); the trend for H tumors to have a higher Ox'-E/ER index did not quite reach statistical significance (*P *= 0.07). These positive correlations were in keeping with the GSEA findings in which tumor phenotypes associated with oxidant-responsive gene sets were also similarly associated with proliferation gene expression.

### Pathway analysis of the Ox-E/ER gene signature

**Figure 3:**
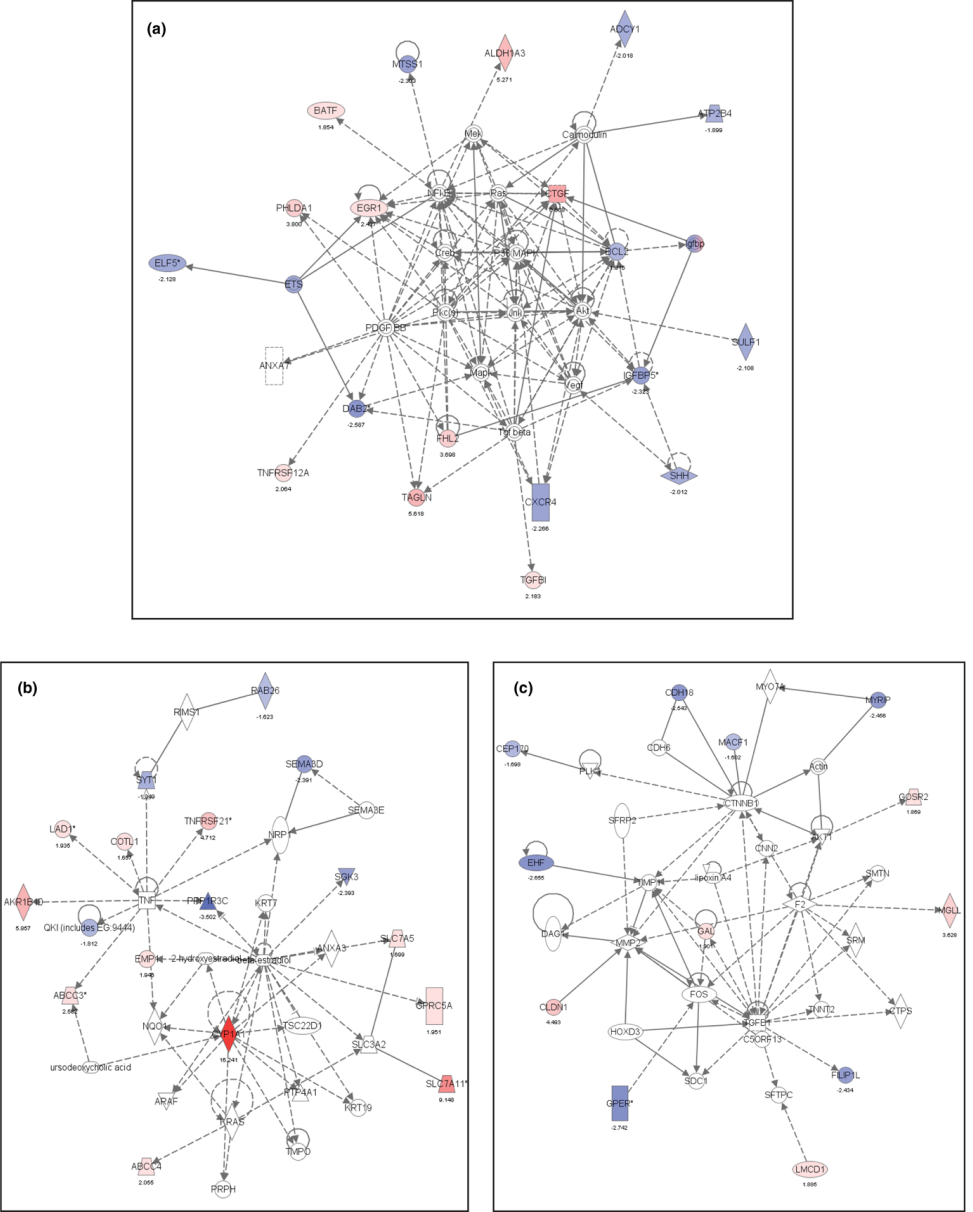
Pathway analysis of the Ox-E/ER gene signature. **(a) **Top scoring gene network represented in the Ox-E/ER gene signature. Ox-E/ER genes upregulated by oxidative stress are colored in red, and oxidative stress downregulated genes are colored in blue. Intensity of color reflects the magnitude of the average fold changes across the three oxidants as listed below the gene. Uncolored nodes are added by the Ingenuity Pathway Systems software, and are not present in the Ox-E/ER signature. Solid lines signify direct gene-gene interactions, whereas broken lines represent indirect relationships that may require secondary effectors not depicted in the network. All connections are supported by at least one published report or from canonical information stored in the Ingenuity Pathway Knowledge Base. **(b) **Number 2 scoring network represented in the Ox-E/ER gene signature. **(c) **Third top scoring network represented in the Ox-E/ER gene signature. ER, estrogen receptor; Ox-E/ER, oxidant-sensitive estrogen/ER gene signature.

The Ox-E/ER gene signature (Table 3) was selected for further functional annotation and prognostic validation based on its robust correlation with clinical PR status and tumor proliferation. Pathway analysis was performed using Ingenuity Pathway Systems, which identified three top networks represented within the 62-gene Ox-E/ER signature. The highest scoring network related to cancer, cell growth, and death, linking 19 Ox-E/ER genes to well defined oxidative stress response pathways such as nuclear factor-κB and c-Jun amino-terminal kinase activation (Figure 3a). Coordinate expression of nine Ox-E/ER genes known to vary in response to platelet-derived growth factor-BB and transforming growth factor-β implicated activated signal transduction downstream from these two breast cancer growth factors. A second network related to cancer and cellular development linked 17 members of this signature to tumor necrosis factor (TNF)-α and estrogen (Figure 3b). The coordinate pattern of expression of eight known TNF-α responsive Ox-E/ER genes reflects downstream effects from this breast cancer cytokine; however, the four estrogen-linked genes did not show coordinate expression consistent with decreased estrogen signaling. A third network linked 12 members of the Ox-E/ER gene signature with cell motility (Figure 3c). Connection nodes of interest included β-catenin (CTNNB1) and matrix metalloproteinase (MMP)2, both of which have previously been correlated to metastasis in various cancers. In particular, increased MMP2 activity has been implicated through direct upregulation by the Ox-E/ER gene CLDN1 [44]. Possible mechanisms connecting alterations in normal β-catenin function through oxidant downregulation of MACF1[45] and CDH18 [46] are also suggested.

**Table 3:** Ox-E/ER gene signature

Affymetrix probe set ID	UniGene symbol	E/ER sensitivity	Oxidant sensitivity	Fold change				
				
				E deprivation	ER-α knockdown	8 hours diamide	8 hours H_2_O_2_	8 hours menadione
213245_at	ADCY1	E/ER Induced	Repressed	0.38	0.46	0.56	0.36	0.57
212136_at	ATP2B4	E/ER Induced	Repressed	0.54	0.52	0.53	0.50	0.55
203685_at	BCL2	E/ER Induced	Repressed	0.25	0.39	0.66	0.43	0.56
213605_s_at	C6orf216	E/ER Induced	Repressed	0.47	0.48	0.53	0.63	0.60
219174_at	CCDC2	E/ER Induced	Repressed	0.49	0.66	0.64	0.61	0.47
206280_at	CDH18	E/ER Induced	Repressed	0.37	0.49	0.45	0.37	0.36
204135_at	DOC1	E/ER Induced	Repressed	0.48	0.50	0.46	0.51	0.26
214240_at	GAL	E/ER Induced	Induced	0.27	0.42	1.84	1.82	2.04
210640_s_at	GPR30	E/ER Induced	Repressed	0.19	0.60	0.46	0.36	0.27
211829_s_at	GPR30	E/ER Induced	Repressed	0.29	0.44	0.43	0.35	0.33
204237_at	GULP1	E/ER Induced	Repressed	0.44	0.56	0.65	0.62	0.49
204235_s_at	GULP1	E/ER Induced	Repressed	0.46	0.64	0.64	0.60	0.48
215913_s_at	GULP1	E/ER Induced	Repressed	0.50	0.64	0.61	0.59	0.53
207719_x_at	KIAA0470	E/ER Induced	Repressed	0.59	0.63	0.57	0.61	0.58
207358_x_at	MACF1	E/ER Induced	Repressed	0.52	0.51	0.66	0.61	0.61
203037_s_at	MTSS1	E/ER Induced	Repressed	0.50	0.63	0.59	0.39	0.32
214156_at	MYRIP	E/ER Induced	Repressed	0.40	0.51	0.48	0.38	0.36
212462_at	MYST4	E/ER Induced	Repressed	0.52	0.53	0.63	0.66	0.36
215643_at	SEMA3D	E/ER Induced	Repressed	0.36	0.39	0.51	0.35	0.39
220038_at	SGK3	E/ER Induced	Repressed	0.15	0.34	0.61	0.32	0.32
207586_at	SHH	E/ER Induced	Repressed	0.16	0.32	0.59	0.53	0.37
216504_s_at	SLC39A8	E/ER Induced	Repressed	0.37	0.55	0.66	0.49	0.61
209921_at	SLC7A11	E/ER Induced	Induced	0.37	0.38	1.68	11.09	8.82
217678_at	SLC7A11	E/ER Induced	Induced	0.37	0.52	2.18	13.46	11.81
201195_s_at	SLC7A5	E/ER Induced	Induced	0.35	0.51	1.60	1.72	1.78
212353_at	SULF1	E/ER Induced	Repressed	0.36	0.45	0.52	0.44	0.46
203998_s_at	SYT1	E/ER Induced	Repressed	0.57	0.54	0.52	0.53	0.49
208161_s_at	ABCC3	E/ER Repressed	Induced	7.30	4.63	1.78	2.29	3.11
209641_s_at	ABCC3	E/ER Repressed	Induced	7.91	3.94	1.79	2.50	3.46
203196_at	ABCC4	E/ER Repressed	Induced	3.08	4.16	1.93	1.93	2.31
206561_s_at	AKR1B10	E/ER Repressed	Induced	2.93	3.00	3.26	5.25	9.07
203180_at	ALDH1A3	E/ER Repressed	Induced	9.43	8.26	3.07	5.80	6.94
205965_at	BATF	E/ER Repressed	Induced	2.35	2.02	1.51	1.98	2.07
207996_s_at	C18orf1	E/ER Repressed	Repressed	5.71	2.07	0.49	0.36	0.47
213618_at	CENTD1	E/ER Repressed	Induced	3.27	2.17	1.65	2.18	3.28
218182_s_at	CLDN1	E/ER Repressed	Induced	2.48	2.64	2.77	5.43	5.28
221059_s_at	COTL1	E/ER Repressed	Induced	2.45	1.81	1.56	1.59	1.82
209101_at	CTGF	E/ER Repressed	Induced	8.30	4.15	1.78	3.68	15.15
217028_at	CXCR4	E/ER Repressed	Repressed	3.22	2.82	0.47	0.56	0.30
205749_at	CYP1A1	E/ER Repressed	Induced	3.28	0.55	6.75	8.96	33.02
210757_x_at	DAB2	E/ER Repressed	Repressed	3.25	1.51	0.51	0.60	0.43
201279_s_at	DAB2	E/ER Repressed	Repressed	3.45	1.67	0.47	0.56	0.39
201278_at	DAB2	E/ER Repressed	Repressed	4.26	1.99	0.47	0.53	0.45
201280_s_at	DAB2	E/ER Repressed	Repressed	4.89	2.30	0.42	0.42	0.32
201694_s_at	EGR1	E/ER Repressed	Induced	5.77	4.42	1.73	3.03	2.52
219850_s_at	EHF	E/ER Repressed	Repressed	4.14	3.96	0.43	0.35	0.35
220625_s_at	ELF5	E/ER Repressed	Repressed	2.27	2.12	0.49	0.53	0.39
220624_s_at	ELF5	E/ER Repressed	Repressed	2.38	2.35	0.54	0.53	0.38
201324_at	EMP1	E/ER Repressed	Induced	15.49	8.23	1.55	1.80	2.49
202949_s_at	FHL2	E/ER Repressed	Induced	1.63	1.56	2.15	3.87	5.07
213144_at	GOSR2	E/ER Repressed	Induced	1.81	1.86	1.93	1.95	1.72
212444_at	GPCR5A	E/ER Repressed	Induced	3.73	2.33	1.51	2.51	1.65
203108_at	GPRC5A	E/ER Repressed	Induced	2.46	2.07	1.57	2.58	1.70
203424_s_at	IGFBP5	E/ER Repressed	Repressed	2.81	1.99	0.52	0.48	0.47
211959_at	IGFBP5	E/ER Repressed	Repressed	3.11	2.23	0.48	0.40	0.41
203287_at	LAD1	E/ER Repressed	Induced	1.70	1.86	1.79	1.81	2.20
216641_s_at	LAD1	E/ER Repressed	Induced	1.72	1.94	1.65	1.56	2.21
218574_s_at	LMCD1	E/ER Repressed	Induced	3.47	1.60	1.58	1.64	2.44
209679_s_at	LOC57228	E/ER Repressed	Induced	3.49	2.47	1.82	1.73	2.17
209373_at	MALL	E/ER Repressed	Induced	7.87	5.99	2.21	3.06	4.35
211026_s_at	MGLL	E/ER Repressed	Induced	2.52	2.78	3.26	2.66	4.96
217996_at	PHLDA1	E/ER Repressed	Induced	2.80	3.15	1.67	3.40	6.33
204284_at	PPP1R3C	E/ER Repressed	Repressed	3.24	2.66	0.41	0.27	0.18
212636_at	QKI	E/ER Repressed	Repressed	8.56	5.16	0.60	0.62	0.44
219562_at	RAB26	E/ER Repressed	Repressed	1.99	3.66	0.62	0.59	0.64
215321_at	RPIB9	E/ER Repressed	Repressed	1.72	1.88	0.66	0.46	0.56
213413_at	SBLF	E/ER Repressed	Repressed	6.25	3.13	0.52	0.51	0.53
220979_s_at	ST6GALNAC5	E/ER Repressed	Repressed	2.87	1.55	0.64	0.61	0.66
205547_s_at	TAGLN	E/ER Repressed	Induced	2.67	4.49	4.18	3.94	8.73
201506_at	TGFBI	E/ER Repressed	Induced	4.45	2.24	1.63	2.11	2.81
218368_s_at	TNFRSF12A	E/ER Repressed	Induced	1.91	1.61	1.74	2.14	2.31
218856_at	TNFRSF21	E/ER Repressed	Induced	4.72	2.65	1.69	4.21	5.40
214581_x_at	TNFRSF21	E/ER Repressed	Induced	5.81	3.41	2.08	5.43	6.63
216920_s_at	TRGC2///TRGV9///LOC442532///LOC442670///TARP	E/ER Repressed	Repressed	2.08	1.61	0.63	0.53	0.49
215806_x_at	TRGC2///TRGV9///LOC442532///LOC442670///TARP	E/ER Repressed	Repressed	2.44	1.62	0.65	0.59	0.52

E, estrogen; ER, estrogen receptor

### Numeric Ox-E/ER index, tumor progesterone status, and clinical outcome

**Figure 4:**
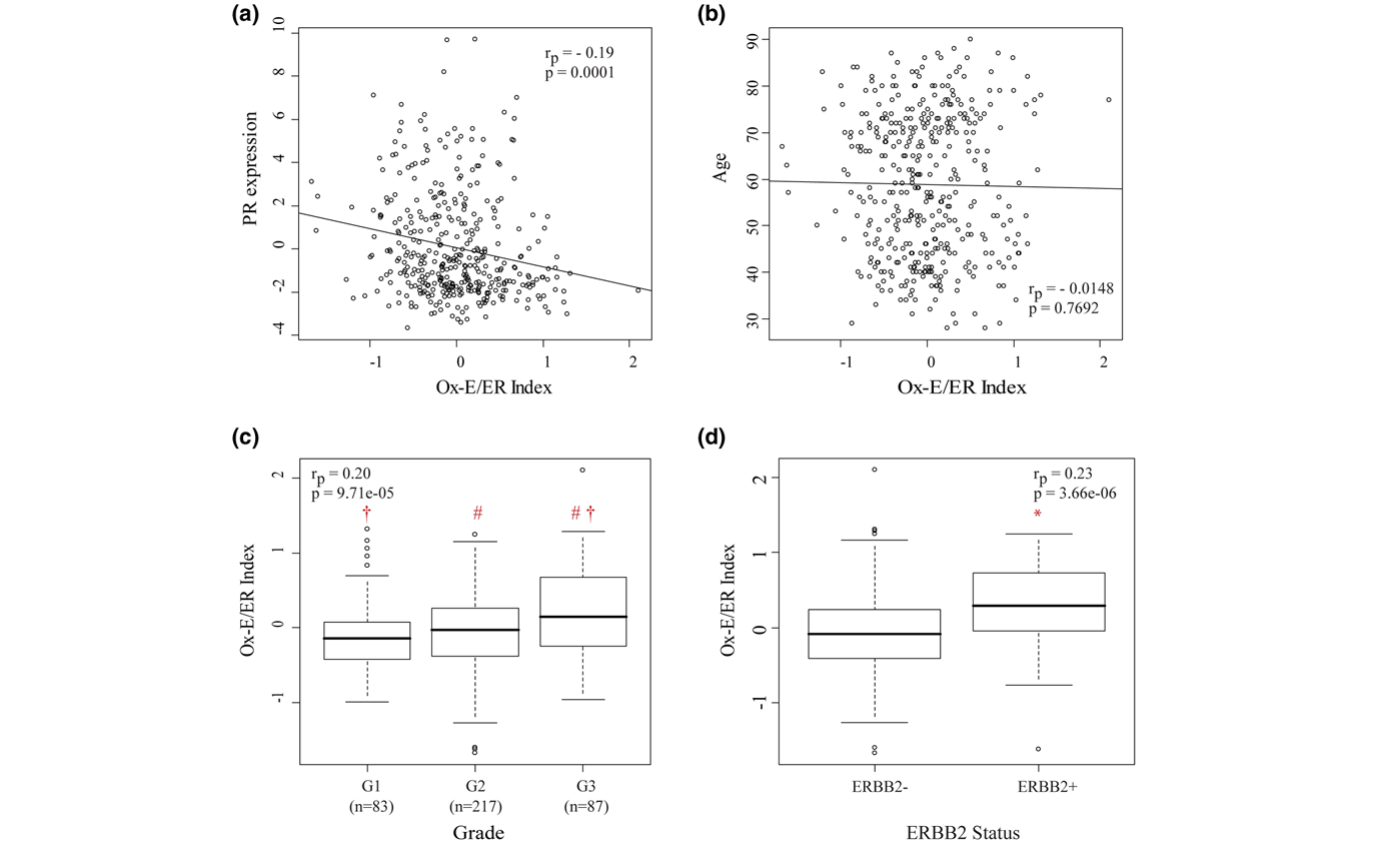
Correlation between Ox-E/ER expression index and clinical parameters in a set of 394 ER-positive breast tumors. **(a) **Scatter-plot of progesterone receptor (PR) expression levels versus Ox-E/ER index values. Line denotes the linear regression fit of the Ox-E/ER index to PR gene expression. **(b) **Scatter-plot of age versus Ox-E/ER index. Line denotes the linear regression fit of the index to age. **(c) **Box plot of the Ox-E/ER index versus tumor grade. Significant differences in Ox-E/ER index with respect to grade are denoted with symbols (# and †; *t*-test, *P *< 0.05). Pearson correlations between the index and grade are also shown. **(d) **Box plot of the Ox-E/ER index versus ERBB2 tumor status, determined as described in Materials and methods. Pearson correlations between the index and ERBB2 status are also shown. *Statistically significant differences in Ox-E/ER index with ERBB2 status (*t*-test, *P *< 0.05). ER, estrogen receptor; Ox-E/ER, oxidant-sensitive estrogen/ER gene signature.

A numeric index calculated from the Ox-E/ER gene expression signature was found to correlate significantly with clinical PR status in the 394 ER-positive primary breast cancers (*t*-test, *P *= 0.0008). The Ox-E/ER signature index was 1.2-fold higher in ER-positive/PR-negative tumors relative to ER-positive/PR-positive tumors and, as shown in Figure 4a, exhibited a significant inverse correlation with PR gene transcript levels (*r*_p _= -0.19; *P *= 0.0001). For comparison, the positive correlation observed between clinical PR status and array determined PR transcript levels from this same dataset was of comparable, albeit slightly greater, magnitude (*r*_p _= +0.24; *P *= 9.033 × e^-07^). In keeping with the GSEA findings, no significant associations were observed between the numeric Ox-E/ER index and age at diagnosis (Figure 4b). Although GSEA failed to detect any significant enrichment of the Ox-E/ER signature genes in ER-positive tumors according to clinical grade, Figure 4c demonstrates that a higher numeric Ox-E/ER signature index correlates significantly with higher tumor grade (*r*_p _= 0.20; *P *= 9.741 × e^-05^), with grade 3 tumors showing an approximately 1.2-fold higher Ox-E/ER index relative to lower grade tumors (*t*-test, *P *= 0.0002 against grade 1 and *P *= 0.001 against grade 2). Also, Figure 4d shows a significant positive correlation between the Ox-E/ER index and array determined ERBB2 over-expression (*r*_p _= 0.23; *P *= 3.66 × e^-06^), with ERBB2 over-expressing tumors exhibiting an approximately 1.3-fold higher Ox-E/ER index than ERBB2-negative tumors.

**Figure 5:**
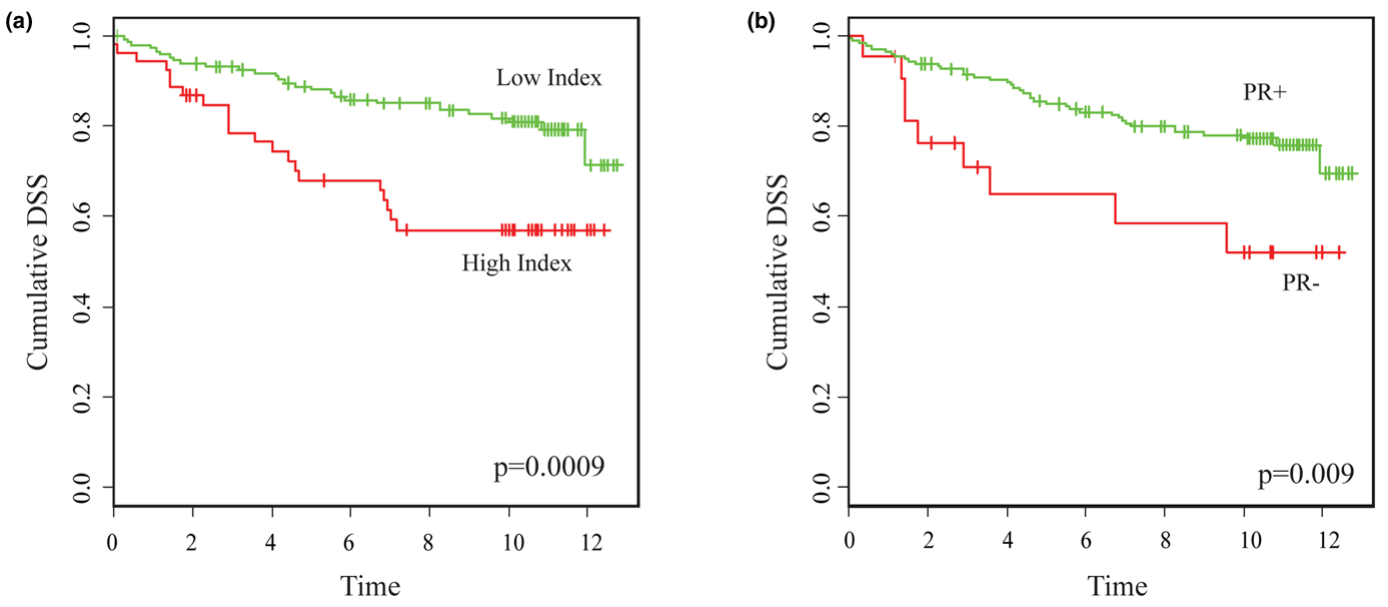
Disease-specific survival analysis with respect to the Ox-E/ER Index and PR status. **(a) **Kaplan-Meier plot of events among the 201 estrogen receptor (ER)-positive cases with known disease-specific survival, dichotomized by high (red) or low (green) Ox-E/ER expression index based on an optimal cut-point determined using an adjusted log-rank statistic. Significance of the difference in survival between groups was determined by log-rank analysis. **(b) **Kaplan-Meier plot of events among the 201 ER-positive cases with known disease-specific survival, dichotomized based on progesterone receptor (PR) status (PR-negative: red and PR-positive: green), with significance determined by log-rank analysis. Ox-E/ER, oxidant-sensitive estrogen/ER gene signature.

Univariate Cox analysis was performed to assess the prognostic value of the numeric Ox-E/ER index in comparison with PR gene expression levels and commonly accepted clinical prognostic parameters including patient age, tumor grade, PR, and nodal status. Except for patient age, the other known prognostic parameters significantly associated with both RFS and DSS. PR expression levels and the Ox-E/ER index both showed strong prognostic trends, with higher PR expression associated with better DSS (*P *= 0.056) and higher Ox-E/ER index tumors associated with worse DSS (*P *= 0.07). Following multivariate Cox analysis, only nodal status retained significance as an independent predictor of either RFS or DSS in this dataset. Restricting further outcome analysis to DSS, an optimized numeric cut-point of 0.3 was defined for the Ox-E/ER index using a modified log-rank statistic. As shown in Figure 5a, this index cut-point dichotomized the 201 ER-positive tumors with DSS data into two different prognostic subgroups: 53 tumors with higher index values and worse DSS, and 148 tumors with lower Ox-E/ER index values and better DSS (log-rank, *P *= 0.000911). Using this optimized cut-point the prognostic value of the Ox-E/ER index appeared to be slightly better than the Figure 5b Kaplan-Meier DSS curve separation achieved using PR status to dichotomize the ER-positive cases (log-rank, *P *= 0.009).

## Discussion

As found over-expressed in human breast cancer cells and targeted by all forms of endocrine therapy, the zinc finger transcription factor ER is sensitive to either reversible or irreversible impairment after even brief exposures to various forms of oxidative stress [18]. Although there have been a number of gene expression studies attempting to identify the suite of estrogen responsive genes expressed in ER-positive human breast cancers [29,47-50], none have yet asked what subset of these estrogen responsive genes are also susceptible to modulation by oxidative stress. To address this question, we performed gene expression microarry studies on the model ER-positive breast cancer cell line MCF7, subjected to either estrogen deprivation or ER-α knockdown, in order to identify a comprehensive set of genes responsive to loss of ER function and sensitive to oxidant stress. Modulation of oxidative stress was accomplished by short-term (8 hours) exposure to one of three different chemical oxidants, each given at a predetermined titre associated with more than 50% loss of intracellular ER transcriptional activity (assayed by transient transfection of on ER element-luciferase reporter gene) without significant loss of ER content or cell viability. Despite the chemical differences between the thiol-specific reactant diamide, the redox-cycling quinone menadione (vitamin K3), and ROS-producing hydrogen peroxide, each of these oxidants has been shown to attack zinc-binding cysteine residues within the ER DNA-binding domain, preventing ER dimerization and direct DNA binding [51,52]. By comparing the differentially expressed genes between control and treatment conditions, we identified 891 estrogen/ER-regulated probes containing a core set of 75 probes (62 unique genes) that were responsive to all three chemical oxidants; these 62 core genes constituted our newly defined Ox-E/ER gene signature. An alternative Ox' signature was experimentally determined by oxidant exposure of MCF7 cells following knockdown of ER-α, from which an alternate Ox'-E/ER signature was derived.

The 891 estrogen/ER-regulated probes exhibited only a 30% overlap with genes previously described as responsive to estrogen stimulation in the same MCF7 cell line model, using the same basic microarray assay platform [29]. Although gene expression changes due to estrogen deprivation have been evaluated in the context of hormone therapy [53-55], neither the effects of estrogen/ER withdrawal nor any comparison of estrogen/ER withdrawal with estrogen-stimulated genes has been reported. False discovery might account for a small fraction of this discrepancy, but the magnitude of difference between the estrogen/ER-responsive gene set identified here and the estrogen-inducible gene set identified previously [29] suggests that estrogen withdrawal and ER loss of function are not entirely reciprocal conditions relative to estrogen stimulation.

Because oxidant-induced loss of ER DNA-binding might not impair all forms of gene regulation by ER (including co-activation of other promoter-bound transcription factor complexes like activator protein-1 and CREB1 [cAMP response element-binding protein 1]), it is notable that 24 out of 27 estrogen/ER-stimulated genes within the Ox-E/ER signature were downregulated by all three oxidants. Because little is known about estrogen/ER-induced gene suppression, in particular its ER DNA-binding requirement, it is not too surprising that only 29 of 48 genes originally suppressed by estrogen/ER were subsequently upregulated by the ER-inactivating oxidant treatments. These observations are consistent with recent genome surveys indicating that ER DNA-binding sites are significantly enriched in the promoters of early estrogen upregulated genes but not enriched in early estrogen suppressed genes [56]. Regarding gene expression differences between the three forms of oxidative stress, the thiol-specific oxidant diamide affected a much smaller subset of estrogen/ER-responsive genes than the less specific oxidants hydrogen peroxide and menadione, both of which are also known to activate kinase pathways. Expression of PR and the estrogen-inducible growth regulator GREB1 were suppressed by hydrogen peroxide and menadione but not by diamide, explaining why these well known ER-associated genes were not part of the 62 Ox-E/ER gene signature and suggesting that their suppression was in part due to oxidant-activated kinase signaling in addition to structural changes in ER. This possibility is supported by previous evidence showing PR downregulation without alteration in ER content or function induced by activation of the phosphoinositide-3 kinase/AKT pathway [8].

The relationships of the five gene signatures identified (E/ER, Ox, Ox', Ox-E/ER, and Ox'-E/ER) to clinical breast cancer cases were explored using data from 394 ER-positive primary human breast cancers pooled from three independently published microarray studies [33-35]. Although PR expression was not commonly affected by all three forms of oxidant treatment, GSEA revealed that oxidant suppressed genes, in particular the oxidant suppressed E/ER regulated genes identified from ER over-expressing MCF7, were suppressed in ER-positive/PR-negative tumors relative to ER-positive/PR-positive tumors, suggesting that loss of PR expression might be a partial surrogate for increased oxidative stress. This suggestion is consistent with our earlier reported correlation between loss of ER DNA-binding in ER-positive/PR-negative breast cancers [20], and with the present observation that numeric Ox-E/ER index values correlate inversely with PR transcript levels and tumor PR status. Recent evidence also indicates that ERBB2 over-expression is associated with loss of PR co-expression in ER-positive breast cancers [57,58]. Although suppression of oxidant-repressed genes was not shown to be associated with ERBB2 over-expression, there was an enrichment of oxidant-induced genes among the ERBB2 over-expressing ER-positive breast tumors, suggesting that elevated oxidative stress is associated with ERBB2 overexpression and may contribute to the loss of PR co-expression seen in ERBB2 overexpressing ER-positive breast cancers.

GSEA comparisons performed using other established gene signatures demonstrated that ER-positive/PR-negative breast cancers express significantly higher levels of proliferation genes relative to ER-positive/PR-positive tumors. Proliferation genes were also expressed at higher levels in the ERBB2 overexpressing breast tumors relative to ERBB2-negative tumors. The observed associations between tumors bearing higher proliferation and oxidative stress signatures are consistent with numerous past observations that mitogenic signaling pathways generate and require increased ROS. Quite consistently, when the oxidant stress gene signatures were considered as numeric indices, these oxidant stress indices were significantly higher in those ER-positive tumors showing higher proliferation gene expression, including ER-positive/PR-negative tumors.

With regard to the putative relationships between aging, breast cancer incidence, and oxidative stress, our GSEA findings were somewhat unexpected in that they demonstrated a weak inverse relationship between oxidative stress and age at diagnosis, suggesting a decoupling of oxidative stress from cellular aging mechanisms that are thought to contribute to breast cancer development. Both GSEA and the Ox-E/ER numeric index also associated oxidative stress with breast tumors of higher grade, a pathologic score strongly influenced by mitotic index. The correlations between oxidative stress and tumor proliferation provide a potential explanation for the apparent decoupling of oxidative stress from aging and breast cancer development, because breast tumors arising earlier in life are known to be more proliferative [33] and to have higher growth rates [59]. Therefore, early age onset breast cancers are probably subject to greater oxidative stress because of their greater proliferative activity.

Focusing on the 62-gene Ox-E/ER signature, which best correlated with clinical PR status in the pooled set of 394 ER-positive breast cancer tumors, Ingenuity Pathway Systems analysis highlighted three top networks relating to cancer, cell development, and cell motility. In accordance with studies associating oxidative stress with kinase signaling [21,60,61], the top scoring network contained 19 of the Ox-E/ER genes linked through various kinases and growth factors as well as two stress-activated transcription factors nuclear factor-κB and CREB1 (cAMP response element-binding protein 1). Of particular interest is this network's inclusion of TGF-β and platelet-derived growth factor-BB, two growth factors extensively evaluated for their involvement in breast cancer progression and metastasis [62-64]. A closer look at the eight Ox-E/ER genes linked to these growth factors revealed that most (EGR1 [65], PHLDA1 [66], IGFBP5 [67,68], TAGLN [68], DAB2 [69], and FHL2 [70]) have been associated with breast cancer. In particular, downregulation of IGFBP5 and upregulation of TAGLN, as observed following oxidant treatment, have been implicated in tamoxifen resistance in a mouse mammary xenograft model [68], suggesting that components of this Ox-E/ER signature may be clinically relevant to the variable endocrine responsiveness of ER-positive breast cancers.

Given that clinical studies have been unable to show why ER-positive/PR-negative breast cancers are significantly more resistant to tamoxifen and other hormonal therapies [4,11], our observations linking the Ox-E/ER signature with both the ER-positive/PR-negative clinical phenotype and a preclinical model of tamoxifen resistance provides new insight into how oxidative stress may contribute to the development of clinically more aggressive forms of ER-positive breast cancers. Of the 16 Ox-E/ER genes involved in the second highest scoring network identified by Ingenuity, nine show a coordinated expression pattern signifying activated TNF signaling. Because oxidative stress is a known byproduct of TNF activation [71], it may not be coincidental that several members of the Ox-E/ER gene signature are downstream targets of activated TNF signaling. Contained in the 11 Ox-E/ER genes that are involved in the third network involving cell motility pathways are two metastasis associated genes, namely MMP2 and CTNNB1 (β-catenin). Although the cell motility pathway was the smallest of the three Ox-E/ER gene networks identified by Ingenuity analysis, it appears to correspond to the only over-represented molecular function linked to the Ox-E/ER gene signature by Gene Ontology analysis in DAVID (Database for Annotation, Visualization, and Integrated Discovery; set size > 5, Expression Analysis Systematic Explorer score < 0.05) [72], that of 'actin-binding' genes.

The numeric Ox-E/ER index allowed for its prognostic evaluation as either a continuous variable or as a categorical parameter, based on an optimized cut-point determination and dichotomization of the pooled ER-positive tumors into those with high versus low Ox-E/ER index values. Despite its correlation with proliferation genes, loss of PR expression, ERBB2 over-expression, and higher tumor grade, the Ox-E/ER index as a continuous variable did not achieve (although it exhibited a trend toward) significance with respect to DSS. However, when an optimized cut-point for the index value was determined (based on a maximized adjusted log-rank statistic) to achieve the greatest possible separation between the Kaplan-Meier DSS curves, the Ox-E/ER index proved capable of dichotomizing the pooled ER-positive breast cancers into two groups whose significant difference in survival exceeded that achieved by PR status alone. This suggests that oxidative stress, and its effects on E/ER signaling, contributes to the development of an aggressive subset of primary ER-positive breast cancers.

## Conclusion

Using a well established cell line model of ER-positive human breast cancer subjected to conditions simulating loss of ER function and oxidative stress, we identified a signature set of 62 unique Ox-E/ER genes, 46 of which are connected within networks associated with increased oxidant signaling, cancer growth factor, and cell motility pathways. Clinical evaluation of this Ox-E/ER signature, as well as alternative oxidant signatures, was performed using a pooled dataset of 394 ER-positive breast cancer cases for which microarray data were available. Despite reports attributing organismal aging to accumulated oxidative stress, there was no evidence to indicate excess oxidative stress in breast cancers arising later in life. To the contrary, the present study indicates that breast cancers showing greater proliferative activity, such as those arising earlier in life, exhibit the greatest oxidative stress. The 62-gene Ox-E/ER signature inversely correlated with tumor PR status and mRNA levels, and was positively associated with ERBB2 over-expression. An optimized Ox-E/ER index cut-point was shown to be capable of dichotomizing the pooled ER-positive breast cancer cases into subgroups whose significant difference in DSS exceeded that achieved by PR dichotomization. Thus, these basic and clinical findings suggest that oxidative stress may contribute to the development of an aggressive subset of primary ER-positive breast cancers, including those exhibiting the ER-positive/PR-negative clinical phenotype.

## Abbreviations

DMEM = Dulbecco's modified Eagle's medium; DSS = disease-specific survival; ER = estrogen receptor; FWER = family-wise error rate; GSEA = gene set enrichment analysis; MAPK = mitogen-activated protein kinase; MMP = matrix metalloproteinase; Ox-E/ER = oxidant-sensitive estrogen/ER gene signature; PR = progesterone receptor; RFS = relapse-free survival; ROS = reactive oxygen species; siRNA = small-interfering RNA; TNF = tumor necrosis factor.

## Competing interests

The authors declare that they have no competing interests.

## Authors' contributions

CY conducted the expression array studies, collated all data, performed biostatistical and informatic analyses, interpreted all results, generated all figures and tables pertaining to the expression array studies, and produced a preliminary draft of the manuscript. CCB conceived the study design, coordinated the RNA studies, formulated all conclusions, and drafted the final manuscript.

## Supplementary Material

Additional file 1 A PDF file showing oxidant treatment effects on cell viability, ER content, and ER transcriptional activity. Methods for the measurement of cell viability, ER content, and transcriptional activation activity at the selected concentration of each oxidant (diamide, H_2_O_2_, and menadione [K3]) are detailed, with pertaining results shown in Figures S1 to S3. Click here for file

Additional file 2 An Excel file showing gene sets used for GSEA. All gene sets were mapped to corresponding Unigene symbols for input into GSEA software. Click here for file

Additional file 3 An Excel file providing a list of genes responsive to each of the eight different MCF7 treatment conditions (72 hours of estrogen [E] deprivation; 72 hours of ER-α siRNA knockdown; 8 hours of diamide; 8 hours H_2_O_2 _and 8 hours of menadione [K3]; 72 hours of ER-α siRNA and 8 hours diamide; 72 hours ER-α siRNA and 8 hours of H_2_O_2_; 72 hours of ER-α siRNA and 8 hours K3). Click here for file
