# Supplementary material for: Genes responsive to both oxidant stress and loss of estrogen receptor function identify a poor prognosis group of estrogen receptor positive primary breast cancers
Source: Breast Cancer Res. 2008 Jul 17;10(4):R61. doi: 10.1186/bcr2120 (PMC2575534; doi:10.1186/bcr2120)

Supplemental Methods:

Cell Viability Assay: Cells were treated with 275μM diamide, 0.5mM H_2O_2, or 10μM K3 for 8 hours. Culture media was changed following treatment and fresh media containing 0.5mg/ml 3-(4,5-dimethylthiazol-2-yl)-2,5-diphenyltetrazolium bromide (MTT) (Sigma, MO) was added. The plate was incubated for four hours at 37°C and 5% CO_2. Media was removed and 100μl of 2-propanol was added. The plate was covered in foil and incubated at room temperature for 15 minutes on a rocking platform. Absorbance at 570nM and 630nM was measured on the spectrophotometer and the reduction number (A_{570}-A_{630}) was recorded. The cell viability was reported relative to the absorbance of the untreated control (viability of control = 1). Statistical significance of changes in cell viability was calculated using t-test ($p<0.05$).

Nuclear Extraction and ERα Immunoblotting: Cells were treated with oxidants for 8 hours at the selected doses and harvested in an NP-40 buffer containing 20mM HEPES, 10mM KCl, 1mM MgCl, 0.5mM DTT, 0.3% NP-40, 20% glycerol and a cocktail of protease inhibitors (Roche Diagnostics, Mannheim, Germany). Cells were disrupted with a dounce homogenizer and centrifuged at 3000rpm for 5min. The nuclear pellet was resuspended in 100μl extraction buffer containing 20mM HEPES, 10mM KCl, 1mM MgCl, 0.5mM dithiothreitol (DTT), 0.1% triton X-100, 20% glycerol, 420mM NaCl and a cocktail of protease inhibitors and placed on a spinning wheel for 20minutes at 4°C. The mixture was centrifuged at 15000rpm for 10minutes at 4°C and the supernatant was collected. Protein aliquots of 5-15μg were separated by gel electrophoresis in 4-12% Bis-Tris gradient genes in MOPS running buffer. Proteins were transferred to PVDF membranes and blocked in 5% non-fat milk in PBS containing 0.1% Tween 20. Membranes were incubated overnight with a mouse monoclonal antibody specific to ERα (Santa Cruz Biotechnology Inc, CA). Immunoreactive bands were visualized with the appropriate horse-radish peroxidase conjugated IgG (BioRad, CA) and chemiluminescence reagents (Pierce, IL). Membranes were then stripped and re-blotted with β-actin antibody (Abcam Inc, MA) for protein loading normalization. Band intensity was quantified using densitrometry.

Plasmid and Transient Transfection: The tk-luc and ERE-tk-luc reporter plasmids were gifts from Dr. Paul Webb. A luciferase reporter driven by the ½ ERE/Sp1 site of the PR-A promoter (+565 ERE/Sp1 TATA-luc), similar to the one used in Petz et al.[1], was created as follows: PGL3-Basic Vector (Promega, MI) was digested with restriction enzymes MluI and BglII (New England Biolabs, MA) at 37 °C for 1 hour. Gel extraction of the digested plasmid was performed using the Qiagen Gel Extraction Kit (Qiagen, CA) as per manufacturer's instructions. Oligo pairs containing the +565 to +602 region of the human PR-A gene promoter and the TATA-box (12bp) from the promoter of the synthetic adenovirus type 12 Elb58-kDa protein flanked by MluI and BglII ends was synthesized (Life Technologies). The oligo pairs were annealed and ligated into the purified digested plasmid with T4 DNA ligase (New England Biolabs, MA) at room temperature for 1 hour. The plasmid was transformed into DH5α strain of *E.coli* (Invitrogen) and purified using the plasmid purification kit (Qiagen, CA) as per manufacturer's protocol.

MCF7 cells were plated at 1000 cells/well in 96-well plates one day prior to transfection. Cells were transiently transfected with 0.1μg of tk-luc, ERE-tk-luc or +565 ERE/Sp1 TATA-luc plasmids per well with the Effectene Transfection Reagent (Qiagen, CA) for 8 hours. Culture media was exchanged and cells were treated with 275μM diamide, 0.5mM H_2O_2, or 10μM K3 for 8 hours. Following oxidant treatment, cells were washed with PBS and luciferase activity was measured using the Luciferase Assay System (Promega, MI). Results were reported as fold changes in luciferase activity over untreated control. Statistical significance of fold changes in luciferase activity were calculated using t-test ($p<0.05$).

Figure S1: Effects of Oxidant Treatment on Cell Viability. Cell viability is plotted as the fraction relative to untreated controls $\pm$ standard error. Each bar represents the average measurement from 8 replicates. * denotes statistically significant difference in cell viability between control and oxidant-stressed samples as measured by the MTT assay. At the concentrations selected, only 275μM diamide treatment produces a small (<20%), but statistically significant, drop in cell viability.

Figure S2: Effects of Oxidant Treatment on ERα content. (A) Western blot of ERα and β-actin following 8h diamide treatments at the indicated doses. The normalized intensity values (ERα/β-actin signal) following treatment relative to untreated control are listed below. (B) Western blot of ERα and β-actin following 8 h H_2O_2 treatments at the indicated doses. The normalized intensity values (ERα/β-actin signal) following treatment relative to untreated control are listed below. (C) Western blot of ERα and β-actin following 8h K3 treatments at the indicated doses. The normalized intensity values (ERα/β-actin signal) following treatment relative to untreated control are listed below.

Figure S3: Effects of Oxidant Treatment on ER Transcriptional Activation. The relative luciferase activity to untreated controls following oxidant treatment $\pm$ standard deviation is plotted. For each luciferase construct, * denotes statistically significant difference in cell viability between control and oxidant-stressed samples as measured by the MTT assay. At the selected concentration, each oxidant produces statistically significant decreases in activation of ERE-TK and ½ ERE-Sp1 driven promoters, without significant changes in the TK-luc activity.

1. Petz LN, Nardulli AM: **Sp1 Binding Sites and An Estrogen Response Element Half-Site Are Involved in Regulation of the Human Progesterone Receptor A Promoter**. *Mol Endocrinol* 2000, **14**(7):972-985.

Figure S1

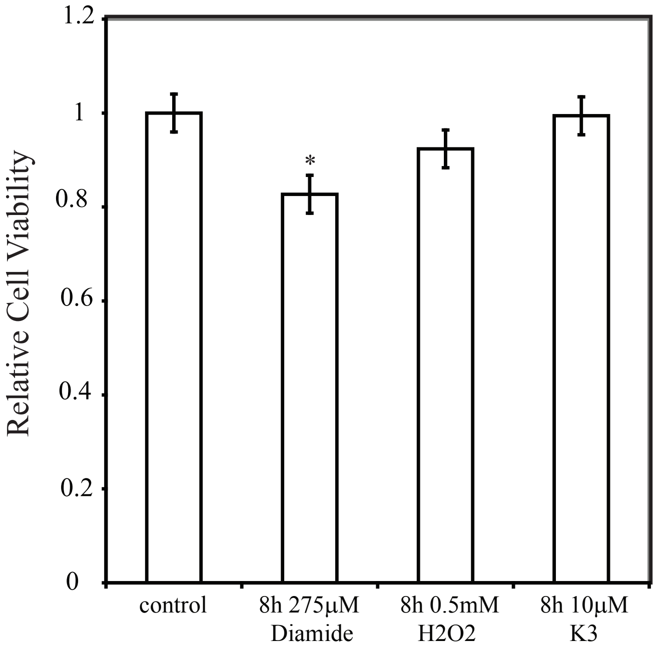

Figure S2

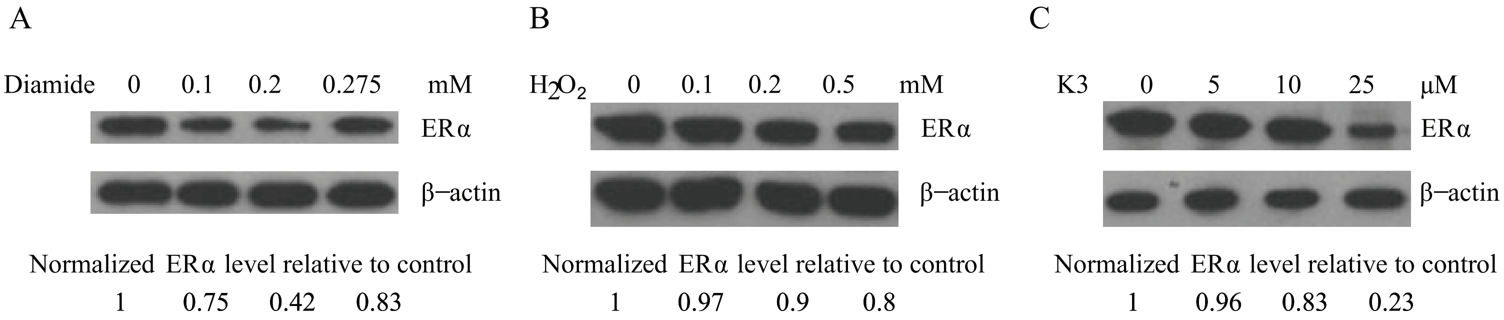

Figure S3

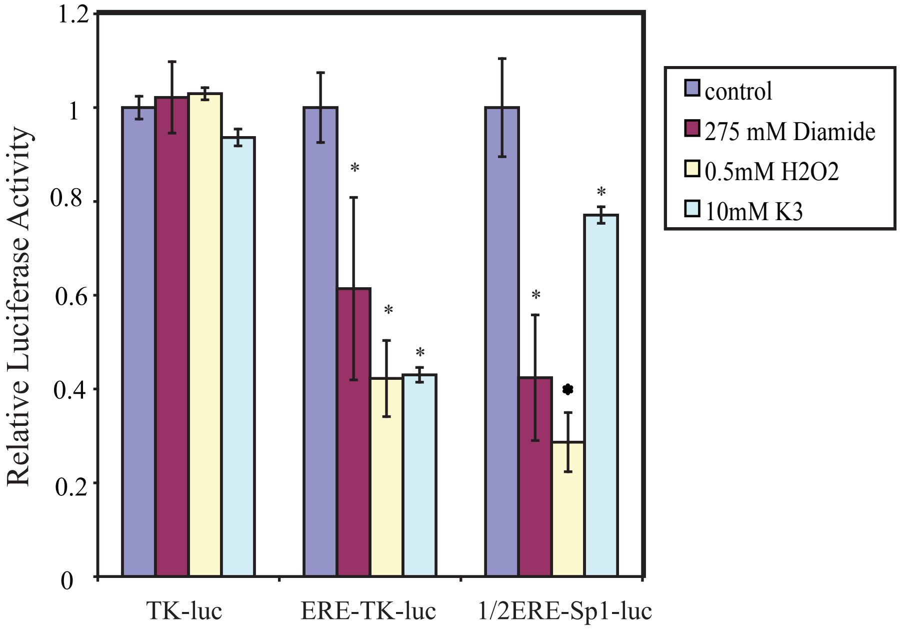

Supplement: Additional file 1 — A PDF file showing oxidant treatment effects on cell viability, ER content, and ER transcriptional activity. Methods for the measurement of cell viability, ER content, and transcriptional activation activity at the selected concentration of each oxidant (diamide, H2O2, and menadione [K3]) are detailed, with pertaining results shown in Figures S1 to S3. [file bcr2120-S1.pdf]
